# Supplementary figures and images for: In silico identification of sugarcane (Saccharum officinarum L.) genome encoded microRNAs targeting sugarcane bacilliform virus
Source: PLoS One. 2022 Jan 20;17(1):e0261807. doi: 10.1371/journal.pone.0261807 (PMC8775236; doi:10.1371/journal.pone.0261807)

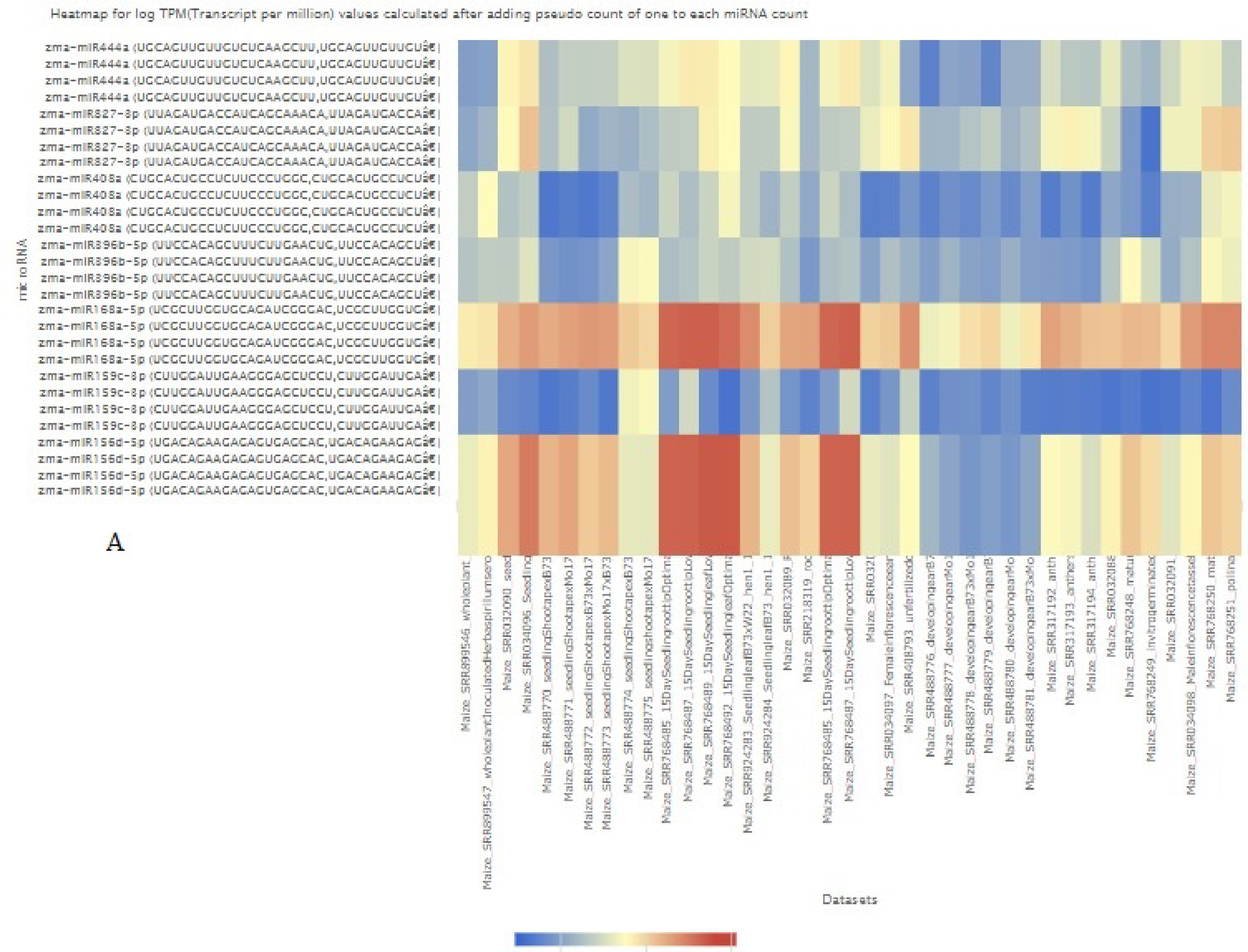

Supplement: S1 Fig — (TIF) [file pone.0261807.s002.tif]

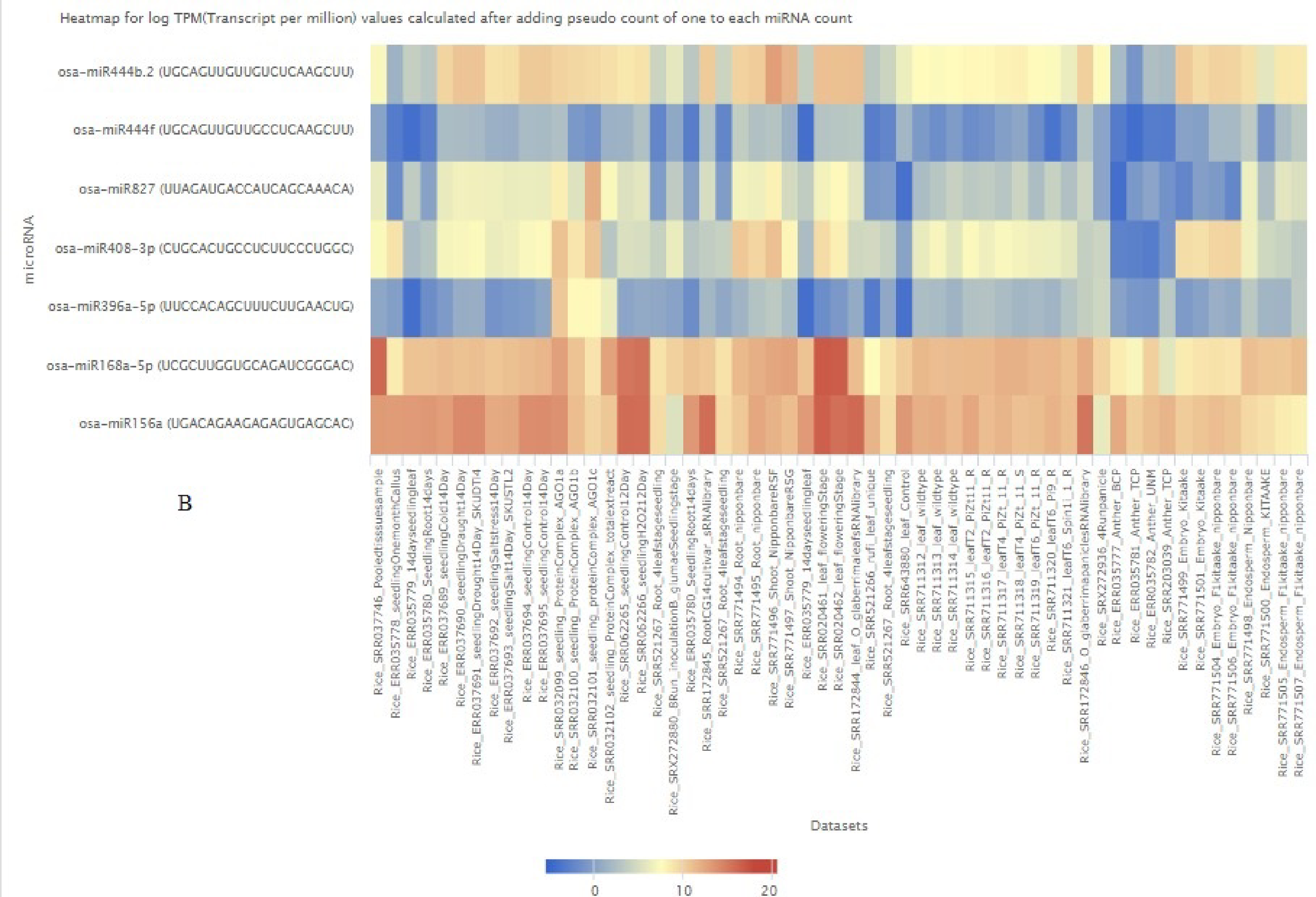

Supplement: S2 Fig — (TIF) [file pone.0261807.s003.tif]

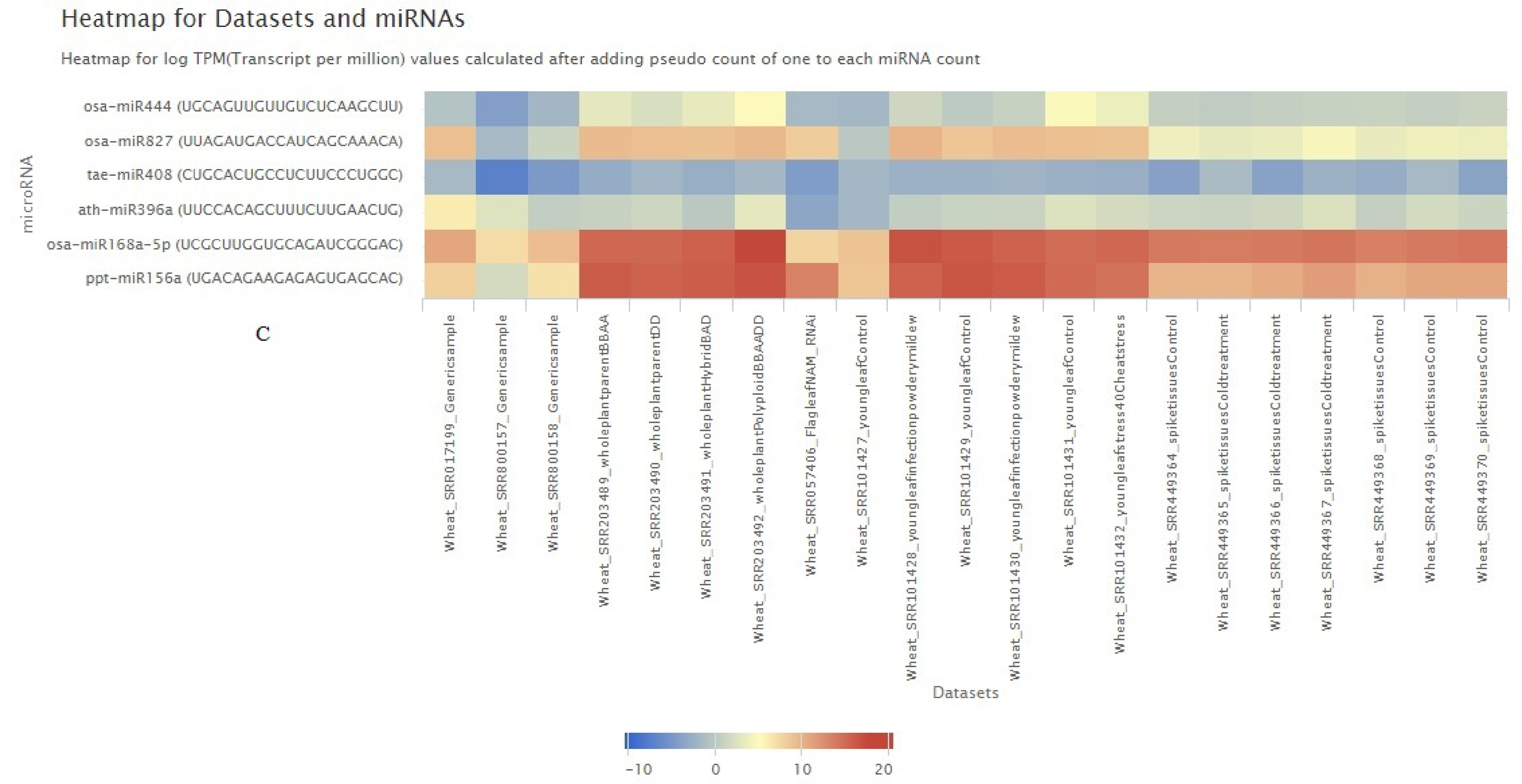

Supplement: S3 Fig — (TIF) [file pone.0261807.s004.tif]
