# Supplementary material for: In silico identification of sugarcane (Saccharum officinarum L.) genome encoded microRNAs targeting sugarcane bacilliform virus
Source: PLoS One. 2022 Jan 20;17(1):e0261807. doi: 10.1371/journal.pone.0261807 (PMC8775236; doi:10.1371/journal.pone.0261807)
Supplement: Attachment — Submitted filename: Response to Reviewers.pdf [file pone.0261807.s006.pdf]

<u>Response to Reviewer’s Comments</u>

Dear Editor,

Coauthors and I very much appreciated the encouraging, critical and constructive comments on this manuscript by the reviewer. The comments have been very thorough and useful in improving the manuscript. We strongly believe that the comments and suggestions have increased the scientific value of revised manuscript by many folds. We have taken them fully into account in revision. We are submitting the corrected manuscript with the suggestion incorporated the manuscript. The manuscript has been revised as per the comments given by the reviewers, and our responses to all the comments are as follows:

<u>Reviewer #2 (First Round of Revision):</u>

In this study, the authors predicted and comprehensively evaluated sugarcane miRNAs for the silencing of SCBV genome using in-silico algorithms. They used several tools to predict the miRNA targets and identified several potential candidate miRNAs which may be used for the silencing of SCBV.

<u>(Minor Concerns)</u>

However, the authors did not provide the expression of these miRNAs and predict their targets in the host sugarcane genome, which should be considered when selecting siRNA.

<u>Reviewer #2 (Second Round of Revision):</u>

"Author Response- Thank you so much for your comments and compliment. We have taken reviewer’s comment in full consideration and it will be well reflected by the revised version of manuscript. Yes, we have not provided the expression data in this revised manuscript. The expression of targeted miRNAs is in process". **<u>I think the authors did not address my comments.</u>**

Respected Sir,

We have greatly improved the existing manuscript for your kind comment after revision. It has increased the values of the current manuscript many folds. We are all very thankful to your comments.

Response- Thank you very much for your valuable suggestion. As per reviewer suggestion, correction has been made in the revised manuscript. Please see line 192-197 in the methodology section. The results section is greatly improved after revision. Please see line 349-356. The supplementary figures have been added in the manuscript (Figure S1, S2 and S3). The references section is also revised with three new references related to expression. Please see reference no. 49, 53 and 54.
