# Supplementary material for: In silico identification of sugarcane (Saccharum officinarum L.) genome encoded microRNAs targeting sugarcane bacilliform virus
Source: PLoS One. 2022 Jan 20;17(1):e0261807. doi: 10.1371/journal.pone.0261807 (PMC8775236; doi:10.1371/journal.pone.0261807)
Supplement: Attachment — Submitted filename: Response to Reviewers.docx [file pone.0261807.s005.docx]

**Response to Reviewer’s Comments**

Dear Editor,

Coauthors and I very much appreciated the encouraging, critical and constructive comments on this manuscript by the reviewers. The comments have been very thorough and useful in improving the manuscript. We strongly believe that the comments and suggestions have increased the scientific value of revised manuscript by many folds. We have taken them fully into account in revision. We are submitting the corrected manuscript with the suggestion incorporated the manuscript. The manuscript has been revised as per the comments given by the reviewers, and our responses to all the comments are as follows:

**Reviewer #1:**

Ashraf et al., have attempted to contribute in addressing the situation caused by SCBV to sugarcanes by envisaging miRNAs that could be helpful in the hushing of SCBV. This study sounds quite useful to me in terms of the economic burden and plants’ health; however, I have come across quite a few major and minor concerns that must be addressed before the manuscript could be accepted for publication.
**(Major Concerns)**

1. The title of the manuscript doesn’t complement the content. It gives a feeling of some novel algorithmic framework design; however, the study doesn’t directly design or improve any algorithm but just entirely depends on the existing platforms. Please modify the title to avoid misguidance.
   .

**Response-** Thank you so much for your suggestion. As per reviewer suggestion, correction has been made in the revised manuscript and we have modified the title. We have removed words “An Algorithmic framework for” from the title and added “*In silico*”. Please see line 1.

2. There is obstruction in the flow of the Abstract portion, please rearrange the sentences and remove any redundant details. Introduction part, the authors mention names like RNAi technology and amiRNA approach, however, I didn’t find enough detail about these approaches in the text. They should be explained for the reader with citations.

**Response-** Thank you very much for your valuable suggestion. Correction has been made as per reviewer suggestion. Redundant details have been removed. Please see line 21-23. Correction has been made as per reviewer suggestion. The introduction section is greatly improved with new text. Please see 62-67 lines for RNAi and lines 78-82 for amiRNA.

3. Line 135 (Page-7), why the authors maintained the default parameters? What makes them sure that the default parameters are suitable in this particular case, because though the default settings are universally applied in most of the conditions, these algorithms could get tricky in situations and they need to be tuned for a particular case. Is there any citation?

**Response-** Thank you so much for your comment. As per reviewer suggestion, correction has been made in the revised manuscript. It was written wrongly. We have replaced it with ‘standard’ instead of ‘default’. Sorry it type error. Please see line 145 of the revised manuscript.

4. Line 138 (Page-7) RNA22, what does the author mean by highly sensitive? This word doesn’t make sense to me. What’s the difference between a sensitive and an insensitive server?

**Response-** Thank you so much for your suggestion. RNA22 does not rely upon cross-species conservation, is resilient to noise, and, unlike previous methods, it first finds putative microRNA binding sites in the sequence of interest, then identifies the targeting microRNA. As per reviewer suggestion, ‘highly sensitive’ is removed. Please see line 148.

5. Please provide the citations for all the maintained or adjusted parameters in case of every server used.

**Response-** Thank you very much for your valuable suggestion. As per reviewer suggestion, correction has been made in the revised manuscript. We used the server as such.

6. In case of mutated viruses, do the outcomes of this study remain the same? The authors should consider variations in the genome since they could greatly alter the genomic behavior.

**Response-** Thank you very much for your valuable suggestion. As per reviewer suggestion, correction has been made in the revised manuscript. After mutation in SCBV viruses, the results will greatly change but it depends upon the kind of mutation in the sequences.

7. Why the algorithms fail to compute data in most of the cases as can be seen in Table 2? Did the authors try to tune the parameters for computing the data?

**Response-** Thank you so much for your comment. We did not tune any parameters during computing the data. The results are as usual the same as predicted by the algorithms.

8. Discussion portion line 1, authors have mentioned that the performance of the computational algorithms is studied which is contradictory to the methodological section where the algorithms are only used for computing the data, not for evaluating or setting any benchmarks for comparisons. Please correct your statement, and search for similar confusions in the manuscript.

**Response-** Thank you so much for your comments. Correction has been made per reviewer suggestion. We have removed the word “performance” and placed it with ‘effectiveness’ Please see line 346.

9. What are the future prospects of this study? How the results could be considered further in terms of the computational drug design?

**Response-** Thank you so much for your comments. Correction has been made in the revised manuscript. We have already showed text in discussion for future work. Please see line 420-424, and 426-434 of the revised manuscript.

10. Please read the manuscript by a native or at least a proficient English user, as I have encountered multiple errors in the punctuation, indentation, and word choice. I have highlighted few of them in the minor concerns but look for other similar issue thoroughly?

**Response-** Thank you so much for your comment. As per reviewer suggestion, correction has been made in the revised manuscript. The manuscript has now been edited by acquiring English correction services from the Mdpi Experts.

**(Minor Concerns)**

a. Line 16 (P-1), expand the full form of ORFs.

**Response-** Thank you so much for your comment. Correction has been made as per reviewer suggestion. Please see line 15.

b. Line 28 (P-1), “Consequently……mutants faster”, the sentence is grammatically wrong.

**Response-** Thank you so much for your comment. Correction has been made as per reviewer suggestion. Please see lines 26-27.

c. Line 42 (P-2), avoid extra spacing?

**Response-** Thank you so much for your comments. Correction has been made as per reviewer suggestion.

d. Line 42 (P-2), that was composed OR that is composed?

**Response-** Thank you so much for your comment. Correction has been made as per reviewer suggestion. Please see line 41.

e. Line 61 (P-3), Plant employs, the word employ is not suitable here as plants use their internal mechanism of defense, instead change it to “possess” or “enriched with”. Line 104 (Page-5) the sentence has unnecessary detail and causes confusion. Instead, just write that the transcript is published and available via accession no. X, and provide the citation. Words like cloning, submission to NCBI etc. are not needed.

**Response-** Thank you so much for your comments. Correction has been made as per reviewer suggestion. Please see line 61 and 115.

f. Citation for pDRAW32 is missing.

**Response-** Thank you so much for your comments. As per reviewer suggestion, correction has been made in the revised manuscript. Please see line 118.

g. Line 180 (Page-9), R is not a language, it’s a statistical environment. Please correct.

**Response-** As per reviewer suggestion, correction has been made in the revised manuscript. Please see line 191.

h. Discussion section, no detail is given about the future prospects of this work.

**Response-** Thank you so much for your comments. As per reviewer suggestion, correction has been made. Please see lines 393-395, 410-415, 420-424 and 426-434.

i. Language needs to be highly improved.

**Response-** Thank you so much for your comments and compliment. We have taken reviewer’s comment in full consideration and it will be well reflected by the revised version of manuscript. As per reviewer suggestion, correction has been made in the revised manuscript. The manuscript has now been edited by acquiring English correction services from the Mdpi Experts. **Editorial Certificate** is attached along with rebuttal letter. We have corrected all the grammatical mistakes in the revised manuscript.

**Reviewer #2:**

In this study, the authors predicted and comprehensively evaluated sugarcane miRNAs for the silencing of SCBV genome using in-silico algorithms. They used several tools to predict the miRNA targets and identified several potential candidate miRNAs which may be used for the silencing of SCBV.

**(Minor Concerns)**
 However, the authors did not provide the expression of these miRNAs and predict their targets in the host sugarcane genome, which should be considered when selecting siRNA.

**Response-** Thank you so much for your comments and compliment. We have taken reviewer’s comment in full consideration and it will be well reflected by the revised version of manuscript. Yes, we have not provided the expression data in this revised manuscript. The expression of targeted miRNAs is in process.
