# Supplementary material for: In silico identification of sugarcane (Saccharum officinarum L.) genome encoded microRNAs targeting sugarcane bacilliform virus
Source: PLoS One. 2022 Jan 20;17(1):e0261807. doi: 10.1371/journal.pone.0261807 (PMC8775236; doi:10.1371/journal.pone.0261807)
Supplement: Attachment — Submitted filename: Response to Reviewers.docx [file pone.0261807.s007.docx]

**Response to Reviewer’s Comments**

Dear Editor,

Coauthors and I very much appreciated the encouraging, critical and constructive comments on this manuscript by the reviewer. The comments have been very thorough and useful in improving the manuscript. We strongly believe that the comments and suggestions have increased the scientific value of revised manuscript by many folds. We have taken them fully into account in this third round of revision. We are submitting the corrected manuscript with the suggestion incorporated the manuscript. The manuscript has been revised as per the comments given by the reviewers 3 and our responses to all the comments are as follows:

**Reviewer #3 (General Comments):**

**General Comment1:** The reviewer’s comments have been partly addressed. For example, abstract and introduction have been revised but still not up to mark. Kindly revise it.

**Response-** Thank you very much for your valuable suggestion. As per reviewer suggestion, correction has been made in the revised manuscript. Please see lines 14-23, 28-30, 40, 48-51, 62-63, 71-73, 75-77, 80, 86, 89-90 and 92-99.

**General Comment2:** There is still no justification for the default values of parameters for prediction methods.

**Response-** Thank you very much for your valuable suggestion. As per reviewer suggestion, correction has been made in the revised manuscript. Please see lines 377-381.

**General Comment3:** The question "What’s the difference between a sensitive and an insensitive server?" is not answered adequately.

**Response-** Thank you very much for your valuable suggestion. As per reviewer suggestion, correction has been made in the revised manuscript. Please see lines 130-135.

**General Comment4:** In response to comment 8, what other confusing words have been replaced is not mentioned. Confusing/incorrect statements may be grammatically correct but technically incorrect. In the abstract the "effective badnaviral methods" is confusing; I think you mean “effective ant-badnaviral methods”

**Response-** Thank you very much your kind comment. Correction has been made a per reviewer suggestion. Please see line 30.

**General Comment5:** Another concern is the validation of the results, still remains.
**Response-** Thank you very much for your valuable suggestion. As per reviewer suggestion, correction has been made in the revised manuscript. We agree on this concern but as this manuscript is only about screening the best possible microRNAs *In silico* to create virus resistance, the wet lab validation is not claimed by authors.

**General Comment6:** One of the comments for "miRNAs targets in the host sugarcane genome" has not been answered too. The amiRNAs may target even the host genomes, so it is important to avoid targeting host targets which can be harmful to plants and include the ones useful to the plant in fighting the virus.

**Response-** Thank you very much for your valuable suggestion. As per reviewer suggestion, correction has been made in the revised manuscript. The host-delivered miRNAs are retrieved from the same plant and they are already expressing inside the host. These miRNAs are expected not make much damage to the plant. We are expected that the increased in expression in the cell will stop the virus to cause extensive damage.

**General Comment7:** Though language corrections have been made, there is still lot of scope for further improvement:-

**Response-** Thank you very much for your valuable suggestion. As per reviewer suggestion, correction has been made in the revised manuscript.

**Reviewer #3 (Minor Concerns):** Comments and suggestions for improvements:-

**Minor Comment:** The revised title is grammatically incorrect. It should be “In silico identification of sugarcane (Saccharum officinarum L.) genome encoded microRNAs targeting sugarcane bacilliform virus”.

**Response-** Thank you so much for your comments and compliment. We have taken reviewer’s comment in full consideration and it will be well reflected by the revised version of manuscript. Revision has been made. Please see line: 1-2.

**Comment 1:** Why is RNAcofold used for the estimation of free energy (ΔG) associated with miRNA–mRNA interactions, while every target prediction software results give free energy value? Justify this in the manuscript.

**Response-** Thank you so much for your comments and compliment. We have taken reviewer’s comment in full consideration and it will be well reflected by the revised version of manuscript. Revision has been made. Please see lines 198-203.

**Comment 2:** In circos author used only selected sugarcane miRNAs and their SCBV targets obtained from miRanda analysis only. Please explain the criteria behind this selection of miRNAs and why only miRanda was used for this graph generation why not other tools?
**Response-** Thank you so much for your comments and compliment. We have taken reviewer’s comment in full consideration and it will be well reflected by the revised version of manuscript. Revision has been made. Please see lines 289-290 in the revised manuscript.

**Comment 3:** In line number 305 it is stated that “The efficacy of the sof-miR159e target against SCBV was validated by the suppression of RNAi-mediated viral combat through the cleavage of viral mRNA or translational inhibition”. Neither any reference nor any methodology is provided regarding this validation.
**Response-** Thank you so much for your comments and compliment. We have taken reviewer’s comment in full consideration and it will be well reflected by the revised version of manuscript. Revision has been made. Please see lines 328. Reference number 43 has been added.

**Comment 4:** Please justify the prediction of Consensus Secondary Structures in section 3.8 while these structures are not used anywhere to conclude anything.
**Response-** Thank you so much for your comments and compliment. We have taken reviewer’s comment in full consideration and it will be well reflected by the revised version of manuscript. The validation of consensual sugarcane miRNAs was confirmed by the prediction of their stable secondary structures using the RNAfold algorithm. Please see lines 335-336.

**Comment 5:** In section 3.9, it is mentioned that table 3 comprises the free energies (ΔG) of sugarcane miRNAs and were estimated as sof-miR159 (a, b, d) (ΔG: -20.10 kcal/mol). But sof-miR159 (a, b, d) are missing from Table 3.
**Response-** Thank you so much for your comments and compliment. We have taken reviewer’s comment in full consideration and it will be well reflected by the revised version of manuscript. Revision has been made. Please see table3 at line 370.

**Comment 6:** Why union plot and intersection plots are not discussed in length while main results are based on these two findings only?
**Response-** Thank you so much for your comments and compliment. We have taken reviewer’s comment in full consideration and it will be well reflected by the revised version of manuscript. Please see lines 414-421 in the discussion section.

**Comment 7:** In line 365 it is stated that “miRanda and psRNATarget are two powerful plant miRNA prediction algorithms for identifying hybridization sites in a viral genome.” The fact is that miRanda is primarily designed for the prediction of miRNA targets in animals. Please give reference in support of your assertion. Further, these algorithms are designed to predict miRNA targets rather than miRNAs.
**Response-** Thank you so much for your comments and compliment. We have taken reviewer’s comment in full consideration and it will be well reflected by the revised version of manuscript. These lines are removed. Please see lines 398-400 in the discussion section.

**Comment 8:** In line 339 it is mentioned that the top effective candidate sof-miR159e has ΔG: -20.40 kcal/mol. This energy is very high for any significant hybridization of miRNA and mRNA in plants. Please justify with reference.
**Response-** Thank you so much for your comments and compliment. We have taken reviewer’s comment in full consideration and it will be well reflected by the revised version of manuscript. The top effective candidate sof-miR159e was selected on the basis of consensual locus position predicted by at three of the algorithms used in this study.

**Comment 9:** Why two highly different ΔG cut off (-15 and -20) for miRanda and RNAhybrid are used while the whole manuscript is based on the comparison.
**Response-** Thank you so much for your comments and compliment. We have taken reviewer’s comment in full consideration and it will be well reflected by the revised version of manuscript. Please see lines 382-386.

**Comment 10:** How did a study of Aedes aegypti cellular miRNA and arboviruses become relevant to the present study?
**Response-** Thank you very much for your valuable suggestion. As per reviewer suggestion, correction has been made in the revised manuscript. This information is removed from the discussion section. Please see lines 438-440 and reference 70.

**Comment 11:** It is mentioned that “We have designed an amiRNA-based gene construct,” while it is not mentioned anywhere in the method section.
**Response-** Thank you very much for your valuable suggestion. As per reviewer suggestion, correction has been made in the revised manuscript. Please see lines 460, 462 and 463.

**Comment 12:** Please include a reference for amiRNA’s mechanism of action.
**Response-** Thank you very much for your valuable suggestion. As per reviewer suggestion, correction has been made in the revised manuscript. Please see line 75 and reference 19.

**Comment 13:** The first-time usage of S. officinarum must be in expanded form at the first instance.
**Response-** Thank you very much for your valuable suggestion. As per reviewer suggestion, correction has been made in the revised manuscript. Please see line 19.

**Comment 14:** The full-length transcript of the SCBV-BRU genome with accession no. JN377537 is used as per the manuscript, then the title of section 3.1. Genome Assembly of SCBV is misleading it should be “Genome Organization of SCBV”.
**Response-** Thank you very much for your valuable suggestion. As per reviewer suggestion, correction has been made in the revised manuscript. Please see line 214.

**Comment 15:** In line 305 it is stated that “The efficacy of the sof-miR159e target against SCBV was validated by the suppression of RNAi-mediated viral combat through the cleavage of viral mRNA or translational inhibition.” However, no reference is provided in this regard.
**Response-** Thank you very much for your comment. Correction has been made as per reviewer suggestion. Reference has been added. Please see line 328 and reference no. 43.

**Comment 16:** Description of tools is repetitive in the manuscript. Firstly in the method section and then in the discussion, the same lines seems redundant.
**Response-** Thank you very much for your valuable suggestion. As per reviewer suggestion, correction has been made in the revised manuscript. Repetitive information and references regarding tool is removed. Please see line 379-382, 388-390 and 394-395.
