# Supplementary material for: In silico identification of sugarcane (Saccharum officinarum L.) genome encoded microRNAs targeting sugarcane bacilliform virus
Source: PLoS One. 2022 Jan 20;17(1):e0261807. doi: 10.1371/journal.pone.0261807 (PMC8775236; doi:10.1371/journal.pone.0261807)
Supplement: Attachment — Submitted filename: Rebuttal Letter.docx [file pone.0261807.s008.docx]

**Response to Reviewer’s Comments**

Dear Editor,

We are grateful to the reviewers and respected editor for their insightful comments on my papers. Coauthors and I very much appreciated the encouraging, critical and constructive comments on this manuscript by the respected editor. The comments have been very thorough and useful in improving the manuscript. We strongly believe that the comments and suggestions have increased the scientific value of revised manuscript by many folds. We have taken them fully into account in this third round of revision. We are submitting the revised manuscript with the suggestion incorporated the manuscript. We have been able to incorporate changes to reflect most of the suggestions provided by the reviewers. We have highlighted the changes within the manuscript. Here is a point-by-point response to the reviewers' comments and concerns. The manuscript has been revised as per the comments given by the respected editor and our responses to all the comments are as follows:

**Editor (Optional Comments): Major Concerns**

**General Comment1:** *No experimental evidence (wet lab) to prove the expression status of select or putatively antiviral miRNAs during viral infection. Had the expression data of sugarcane miRNAs was used in the analysis it would have been still acceptable. However, that was not the case.*

**Response-** Thank you so much for your comments and compliment. We have taken reviewer’s comment in full consideration and it will be well reflected by the revised version of manuscript. Revision has been made previously and accepted by the reviewer 2. Please see lines: 193-198 and 351-358.

**General Comment2:** *Experimental evidence for the expression of host miRNAs along with RACE experiments to prove the cleavage of viral mRNA is a must to claim the antiviral role of host miRNAs. In the absence of any evidence it is a mere prediction which may or may not work.*

**Response-** Thank you very much for your valuable suggestion. Thank you for this suggestion. It would have been interesting to explore this aspect. However, in the case of our study, it seems slightly out of scope and is part of our research in future.

**General Comment3:** *When the study predicts that a select list of host miRNAs could target the viral genome sequence (or ORFs) the need for artificial miRNA construct in conferring virus resistance has not explained properly. Quite interestingly authors claim they have modified the miRNA/miRNA* sof-miRNA159. However what is the necessity for this modification or kindly explain what has been modified adequately.*

**Response-** Thank you very much for your valuable suggestion. As per reviewer suggestion, correction has been made in the revised manuscript. There is no modification. We have removed the figure 10. Please see lines 437-440 and 449-453.

**General Comment4:** *The last sentence of the abstract states the following [The efficacies of the predicted candidate miRNAs are evaluated here to test the survival rates of the in vitro amiRNA-mediated effective anti-badnaviral methods in terms of silencing and resistance in sugarcane cultivars] however there are no experimental evidence to prove the efficacy of miRNAs in vitro or in vivo in silencing badnavirus genome.*

**Response-** Thank you very much for your valuable comment for the improvement of the manuscript. Correction has been made as per editor suggestion. Please see lines 28-31.

**General Comment5:** *A greater portion of MS is wasted in explaining the features of miRNA target prediction algorithms. Specific web links to the algorithms could suffice for the reader to understand what these softwares do.*

**Response-** Thank you very much for your valuable comment for the improvement of the manuscript. Thank you for this suggestion. It would have been interesting to explore this aspect. Appropriate changes were made and highlighted in the revised manuscript according to the suggestions of the respected editor. We agree this and have incorporated your suggestion throughout the manuscript. Please see lines 138-139, 141-143, 149-153, 157-158, 161-163,170-174, 182-183 and 185-192.

**Editor Comments: Minor Concerns**

**General Comment1:** *Still manuscript writing is not satisfactory.*

**Response-** Thank you very much for your comments. This manuscript was edited for proper English language, grammar, punctuation, spelling, and overall style by one or more of the highly qualified native English speaking editors at MDPI Journal Experts. The editorial certificate was uploaded with the revised manuscript at first revision stage. I hope the revised manuscript will meet the requirements of academic publishing in PLoS ONE Journal and I have revised my manuscript according to the suggestions of the professional editor. Thanks again for the reminder.

**General Comment2:** *There exists no connectivity whatsoever between what is presented in lines 78-82 and the lines just before (71-78).*
**Response-** The comments from the peer-reviewers are very useful for the improvement
of our manuscript. Correction has been made in the revised manuscript. Please see lines 80-84.

**General Comment3:** *However having said these, the title unequivocally states that the work is an in silico analysis. Hence I would suggest the authors to tone down the use of amiRNA strategies etc in the MS*.

**Response-** The comments from the peer-reviewers are very useful for the improvement
of our manuscript. Correction has been made in the revised manuscript. Please see lines 94-95, 445-447 and 461.

**General Comment3:** *Language needs to be rechecked once again.*

**Response-** The comments from the peer-reviewers are very useful for the improvement
of our manuscript. Thank you very much for your comments. This manuscript was edited for proper English language, grammar, punctuation, spelling, and overall style by one or more of the highly qualified native English speaking editors at MDPI Journal Experts. Thanks again for the reminder. The manuscript will be submitted again for English correction after final acceptance.
