# Supplementary material for: In silico identification of sugarcane (Saccharum officinarum L.) genome encoded microRNAs targeting sugarcane bacilliform virus
Source: PLoS One. 2022 Jan 20;17(1):e0261807. doi: 10.1371/journal.pone.0261807 (PMC8775236; doi:10.1371/journal.pone.0261807)
Supplement: Attachment — Submitted filename: Rebuttal Letter.docx [file pone.0261807.s009.docx]

**Response to Reviewer’s Comments**

Dear Editor,

We are grateful to the reviewers and respected editor for their insightful comments on my papers. Coauthors and I very much appreciated the encouraging, critical and constructive comments on this manuscript by the respected editor. The comments have been very thorough and useful in improving the manuscript. We strongly believe that the comments and suggestions have increased the scientific value of revised manuscript by many folds. We have taken them fully into account in this fourth round of revision. We are submitting the revised manuscript with the suggestion incorporated the manuscript. We have been able to incorporate changes to reflect most of the suggestions provided by the reviewers. We have highlighted the changes within the manuscript. Here is a point-by-point response to the reviewers' comments and concerns. The manuscript has been revised as per the comments given by the respected editor and our responses to all the comments are as follows:

**Editor (Optional Comments): Minor Concerns**

**General Comment1:** *Line NO 79 Experimental validation of potential miRNA targets is highly costly and laborious. Kindly remove it.*

**Response-** Thank you so much for your comments and compliment. We have taken reviewer’s comment in full consideration and it will be well reflected by the revised version of manuscript. Revision has been made. Please see line: 79.

**General Comment2:** *Fig. 1 Title states “The methodology of miRNA prediction from the SCBV genome” Kindly change in to “The methodology of host or sugarcane miRNA target prediction in the SCBV genome”*

**Response-** Thank you very much for your valuable suggestion. Revision has been made. Please see lines: 120-121.

**General Comment3:** *Also the legend says “A flowchart designed for predicting candidate miRNAs from the SCBV genome pipeline”. In fact it is candidate miRNAs of host that could potentially target SCBV Genome. Change this too.*

**Response-** Thank you very much for your valuable suggestion. As per reviewer suggestion, correction has been made in the revised manuscript. Please see lines 121-122.

**General Comment4:** *Kindly consult native English speaker to rectify the errors in the MS.*

**Response- Response-** Thank you very much for your valuable comments for the improvement of our manuscript. This manuscript was edited for proper English language, grammar, punctuation, spelling, and overall style by one or more of the highly qualified native English speaking editors at MDPI Journal Experts two times: First round of revision and final round of revision. The editorial certificates were uploaded with the revised manuscript in the system. I hope the revised manuscript will meet the requirements of academic publishing in PLoS ONE Journal and I have revised my manuscript according to the suggestions of the professional editor. Appropriate changes were made and highlighted in the revised manuscript according to the suggestions of the respected English editor. We agree this and have incorporated your suggestion throughout the manuscript using service of English editor at MDPI experts. We hope the manuscript is now acceptable for publication.

**Journal Requirements:**
Please review your reference list to ensure that it is complete and correct. If you have cited papers that have been retracted, please include the rationale for doing so in the manuscript text, or remove these references and replace them with relevant current references. Any changes to the reference list should be mentioned in the rebuttal letter that accompanies your revised manuscript. If you need to cite a retracted article, indicate the article’s retracted status in the References list and also include a citation and full reference for the retraction notice.

**Response:** Thank you so much for your comments and compliment. We have checked and there is no error found and all references are correct.
